# Interest paradigm for early identification of autism spectrum disorder: an analysis from electroencephalography combined with eye tracking

**DOI:** 10.3389/fnins.2024.1502045

**Published:** 2024-11-27

**Authors:** Binbin Sun, Elombe Issa Calvert, Alyssa Ye, Heng Mao, Kevin Liu, Raymond Kong Wang, Xin-Yuan Wang, Zhi-Liu Wu, Zhen Wei, Xue-jun Kong

**Affiliations:** ^1^Shenzhen Maternity and Child Healthcare Hospital, Shenzhen, China; ^2^Athinoula A. Martinos Center for Biomedical Imaging, Department of Radiology, Massachusetts General Hospital and Harvard Medical School, Boston, MA, United States; ^3^Beth Israel Deaconess Medical Center and Harvard Medical School, Boston, MA, United States

**Keywords:** autism spectrum disorder (ASD), eye-tracking (ET), electroencephalography (EEG), interest paradigm, early diagnosis, biomarker

## Abstract

**Introduction:**

Early identification of Autism Spectrum Disorder (ASD) is critical for effective intervention. Restricted interests (RIs), a subset of repetitive behaviors, are a prominent but underutilized domain for early ASD diagnosis. This study aimed to identify objective biomarkers for ASD by integrating electroencephalography (EEG) and eye-tracking (ET) to analyze toddlers’ visual attention and cortical responses to RI versus neutral interest (NI) objects.

**Methods:**

The study involved 59 toddlers aged 2-4 years, including 32 with ASD and 27 non-ASD controls. Participants underwent a 24-object passive viewing paradigm, featuring RI (e.g., transportation items) and NI objects (e.g., balloons). ET metrics (fixation time and pupil size) and EEG time-frequency (TF) power in theta (4-8 Hz) and alpha (8-13 Hz) bands were analyzed. Statistical methods included logistic regression models to assess the predictive potential of combined EEG and ET biomarkers.

**Results:**

Toddlers with ASD exhibited significantly increased fixation times and pupil sizes for RI objects compared to NI objects, alongside distinct EEG patterns with elevated theta and reduced alpha power in occipital regions during RI stimuli. The multimodal logistic regression model, incorporating EEG and ET metrics, achieved an area under the curve (AUC) of 0.75, demonstrating robust predictive capability for ASD.

**Discussion:**

This novel integration of ET and EEG metrics highlights the potential of RIs as diagnostic markers for ASD. The observed neural and attentional distinctions underscore the utility of multimodal biomarkers for early diagnosis and personalized intervention strategies. Future work should validate findings across broader age ranges and diverse populations.

## 1 Introduction

Autism spectrum disorder (ASD) is a heterogeneous neurodevelopmental condition that manifests through deficits in social communication and the presence of restricted, repetitive behaviors (RRBs) (American Psychiatric Association, 2013) with an estimated global prevalence of 1% (Zeidan et al., 2022). Early intervention is paramount as it plays a crucial role in significantly improving the prognosis of children with ASD as they transition into adulthood. This significance was underscored in a longitudinal study demonstrating that early intervention by parents at ages 2–3 correlates with marked improvements in adaptive skills, intelligence quotient (IQ), and even the likelihood of achieving full independence (Anderson et al., 2014). Thus, finding biomarkers that are highly predictive of ASD is of paramount importance. Early diagnosis and intervention can be facilitated by such biomarkers, enabling timely support and treatment for affected children. Current biomarkers such as those related to visual attention (Bradshaw et al., 2020; Mason et al., 2021) and neurophysiology (Hiremath et al., 2021; Roberts et al., 2021; Shic et al., 2022) predominantly examine the social communication domain of ASD symptomatology, rather than RRBs. Although often overlooked in ASD research, the presence of early onset RRBs can predict the severity and prognosis of ASD (Leekam et al., 2011; Wolff et al., 2014) and thus is a promising domain to study in the search for reliable, predictive biomarkers for ASD diagnosis.

Restricted interest (RI), a subset of the broader category of RRBs, is characterized by intense and narrowly focused interests that often affect daily functioning (Carter et al., 2020). According to South et al. (2005), parents report that the most challenging ASD symptoms to manage daily are those related to rigid, repetitive circumscribed interests. Furthermore, the pervasive nature of these interests contributes to difficulties in social skill acquisition and peer communication with some researchers purporting that RIs are driving the social communication deficit seen in ASD (Zivan et al., 2021). Despite their significant functional impairment, restrictive interests (RIs) are among the most prevalent symptoms in ASD, affecting 75–95% of individuals (Grove et al., 2018). Given their high frequency in ASD and their detrimental impact on social development and functioning, RIs constitute a pertinent area of study for the development of biomarkers to enable early diagnosis and facilitate timely intervention. Thus, in this study, we aim to identify RIs among ASD toddlers with the use of a 24-object passive viewing paradigm. The restricted visual attention of toddlers will be quantified using electroencephalographic (EEG) time-frequency (TF) power and eye tracking (ET) indices including pupil size and fixation time.

EEG measurement has become a vital biological indicator for diagnosing and providing treatment feedback for children and adolescents while serving as an important data source for quantitatively measuring cortical dynamics (Loo et al., 2016). In recent years, TF analysis has received widespread attention in the study of ASD (Levin and Nelson, 2015), revealing how brain signals are composed across various frequencies and time windows, effectively capturing the time dynamic of the three features of neural data: frequency, power, and phase (Morales and Bowers, 2022). This analytical approach provides a new perspective for a deeper understanding of the neural mechanisms of ASD. The EEG-derived measure, TF power, thus has been put forward as a potential biomarker owing to its ability to measure cortical activity (Wang et al., 2013) and thus disruptions in the brain’s oscillatory rhythms (Gabard-Durnam et al., 2019). Early brain activity changes have been demonstrated in ASD, with atypical connectivity noted in various frequency bands across the power spectrum of infants that later go on to develop ASD (Orekhova et al., 2014). This early atypical connectivity occurs on a background of aberrant brain network development that begins early after birth that leads to increased brain growth causing local hyperconnectivity and long distance hypoconnectivity between different brain regions (O’Reilly et al., 2017). The corpus of recent ASD research notes that differences in power across frequency bands can be seen as early as 3 months postnatal, with high-risk ASD infants showing lower alpha and beta power in the frontal cortex (Tierney et al., 2012; Gabard-Durnam et al., 2015; Levin et al., 2017). Furthermore, at 6 months of age, infants at high risk for autism exhibited lower spectral power across delta, theta, low alpha, high alpha, beta, and gamma bands in frontal regions compared to low-risk infants; these high-risk infants also demonstrated distinct trajectories of spectral power changes as they developed. These differences seen in early life have been shown to delineate the core symptom domains of ASD, with evidence from a study of high-risk ASD toddlers expanding on the idea that hyper-alpha connectivity represents an attentional style that is over-focused and more closely related to restricted interests compared to other domains of RRBs (Sasson et al., 2011). Therefore, EEG power spectrum analysis might be a potential asset to be used to examine circumscribed interests in toddlers in the hopes of finding highly predictive biomarkers for ASD.

According to Kim et al. (2022), attention and arousal are regulated by the locus coeruleus (LC) in the brainstem that produces the neuromodulator norepinephrine (NE). According to the adaptive gain theory, the locus coeruleus norepinephrine (LC-NE) system operates in two modes, a phasic and a tonic state (Berridge and Waterhouse, 2003; Poe et al., 2020), which can be monitored indirectly through changes in pupil size (Kim et al., 2022). Pupil dilation studies that have focused on reactions to social stimuli or luminance changes have demonstrated atypical pupil metrics among ASD subjects, indicating LC-NE dysfunction. Therefore, employing ET, specifically pupillometry, can provide insight into LC-NE activity and potentially enhance our understanding of restricted attention in ASD. Furthermore, ET has emerged as a valuable tool in ASD research, offering a non-invasive, convenient, and safe method to study the disorder (Shic et al., 2022). By capturing pupil size, and fixation time metrics, ET technology provides insights into the atypical neural mechanisms underlying ASD and an objective approach for quantifying visual attention. This is particularly important because studies have shown that children with ASD often display differential interest in non-social objects such as cars, trains, and boats (Bodfish et al., 2000; Sasson et al., 2008, 2011). These differences in interest can be quantified, allowing us to better understand RIs observed in ASD and how they may differ from non-ASD subjects, especially in the early stages of development before behavioral atypicalities emerge. As the primary methods for identifying restrictive interests often rely on subjective measures, such as caregiver reports, observation, and questionnaires (Tian et al., 2022), employing objective approaches like ET holds promise in establishing reliable biomarkers for ASD diagnosis.

In our previous study (Sun et al., 2023), we investigated RIs in ASD using a sequential visual paradigm, incorporating EEG functional connectivity, pupil size, and fixation time as indices. Our findings revealed that under high restrictive interest stimulation (HRIS), children with ASD exhibited significantly higher alpha band connectivity, increased fixation time, and pupil enlargement compared to non-ASD children. Furthermore, by employing a network-based machine learning approach, we achieved an area under the ROC curve (AUC) of 0.81 (95% CI: 0.61–0.98) using ADOS-2 scores and fixation time as predictors. Recent autism research has delved into the integration of ET and EEG modalities in studying RRBs, with most studies focusing on paradigms centered around social or facial stimuli (Thapaliya et al., 2018; Geng et al., 2020; Zhang et al., 2021). Notably, Sasson et al. (2008) employed an object-only array to investigate circumscribed interests while using ET to quantify visual attention. However, the integration of TF power, pupil size, and fixation time to examine restricted interests (RIs) in ASD using an object-only viewing paradigm remains unexplored. Therefore, in this current study, we aim to identify RIs in ASD toddlers using a visual paradigm designed to include images of objects known to be of RI (South et al., 2005) and others of neutral interest. Expanding on our prior research, this study explores the use of a single object paradigm instead of a sequential one, allowing us to examine RIs in an environment of competing stimuli, which could reveal nuances underlying RIs in ASD. In addition, early abnormalities in TF power, particularly observed in high-risk ASD infants (Tierney et al., 2012; Gabard-Durnam et al., 2015; Levin et al., 2017) present an opportune avenue for early detection and thus will be explored in this current work. Therefore, by leveraging EEG TF power and ET metrics—specifically, pupil size and fixation time—we aim to detect increased responses to RI objects within the ASD group. We intend to integrate indices from both modalities and construct logistic regression models to predict ASD as an outcome.

## 2 Materials and methods

### 2.1 Participants

Participants comprised 32 children diagnosed with ASD, aged 2–4 years, and 27 age and sex-matched non-ASD controls. The diagnosis for ASD participants was established according to the Diagnostic and Statistical Manual of Mental Disorders, Fifth Edition (DSM-5) and the Autism Diagnostic Observation Schedule, Second Edition (ADOS-2), with an additional requirement of a Childhood Autism Rating Scale (CARS) score above 30. Non-ASD controls were screened to exclude psychiatric and neurological disorders, including ASD and developmental delay. Exclusion criteria for all participants included hyperactivity, refusal to wear an electrode cap, and astigmatism. Participants were not on medication for at least 2 weeks before EEG recording and washed their hair the day before participating in the study to reduce scalp oil interference. Ethical approval was granted by the Ethics Committee of Shenzhen Maternity and Child Healthcare Hospital (ID: SFYLS 2022), with informed consent obtained from the guardians of all participants.

### 2.2 Experiment stimulus

**Figure 1:**
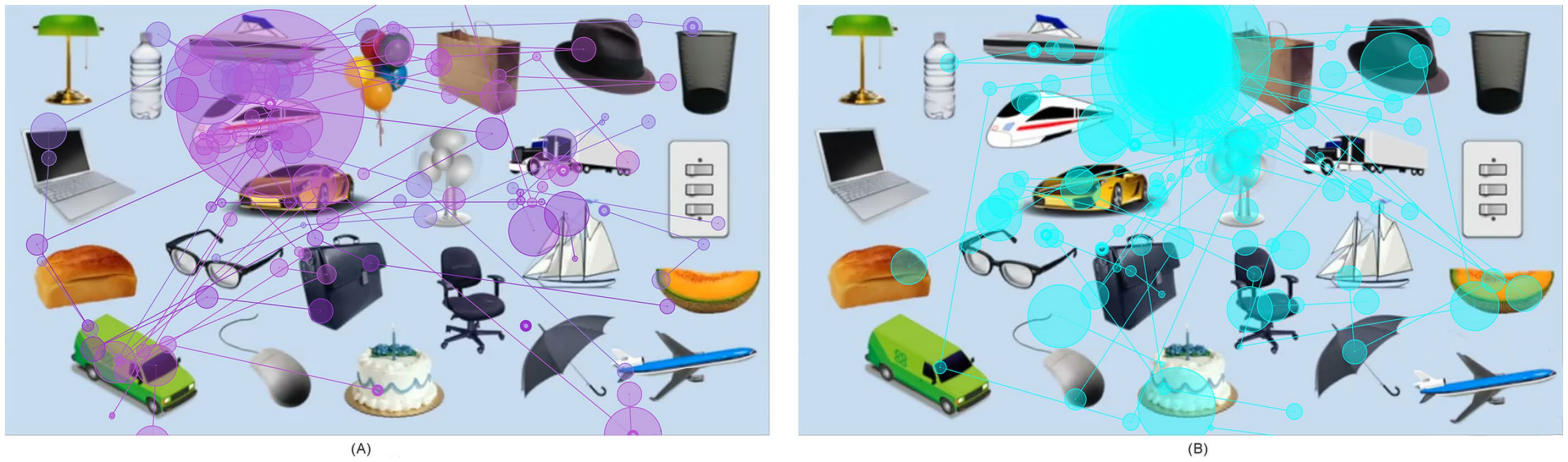
Heat maps of eye tracking fixation times for viewing the interest paradigm. (A) Fixation trajectory and fixation times of children in the ASD group. (B) Fixation trajectory and fixation times of children in the non-ASD group. The connected purple and blue lines represent eye-tracking fixation trajectories, and the purple circle and the blue circle represent the heat map of the fixation times. The larger the circle and the darker the color, the longer the fixation times.

An object-only interest paradigm was created based on the ASD Stereotyped Behavior Scale and the Yale Special Interest Interview (South et al., 2005). This paradigm comprised 24 objects, which are categorized into restricted interest (RI) and neutral interest (NI) objects (see Figure 1). The RI objects included transportation vehicles like cars, planes, bullet trains, and electronic products such as computers and a mouse. Additionally, NI objects like balloons, cakes, switches, and hats representing daily life items were included. Objects directly associated with social attributes, such as faces and eyes, were not included. Standardization of the image size (360 × 864 pixels) and resolution (5 × 12 inches) was achieved using Adobe Photoshop CC 2021.^1^

### 2.3 Experimental process

Data collection for our experiments employed the EyeLink 1000 Plus Eye Tracker alongside a 32-lead EEG system (HydrocCel Geodesic Sensor Net and a Net Amps 300 amplifier) produced by SR Research and Electrical Geodesics, Inc. (EGI) respectively. Our experiments were conducted in a sound-controlled examination room free from electromagnetic interference that had constant lighting. Parents holding study participants were positioned 65 cm away from the eye tracker display, with the subject’s chin fixed on the chin rest to maintain head stability. The settings of the setup were calibrated to optimize eye tracking and the recording of EEG parameters, with a sampling rate of 1,000 Hz, pupil size resolution 0.2% of diameter, and using a sampling rate of the pupil and cornea (pupil + CR) in both eyes, so that the sampling cursor for both eyes turn green at the same time. The specific parameters are left eye, error: <0.5° avg., <1° max (POOR), right eye, error: <0.5° avg., <1° max (POOR), with the right eye being selected as dominant. The sampling rate was set to 250 Hz and the Cz electrode as the reference, with the impedance of all electrodes kept at less than 50 kΩ. The procedure was explained to the participants and their guardians before commencement to ensure comfort and comprehension. At the beginning of the experiment, the researcher guided the participants to complete the five-point calibration of the right eye and then instructed the participants to view the 24-object interest paradigm. The entire experiment lasted for 2–5 min. Additional details on the experimental procedure, including calibration and trial timing, are provided in Supplementary File 1.

### 2.4 ET data preprocessing

The preprocessing of raw pupil size data was performed using MATLAB2019.^2^ Firstly, trials with a single blink duration exceeding 100 ms were eliminated, while those with blinks lasting less than 100 ms underwent linear interpolation to sample and interpolate missing points, thereby maintaining data integrity. Additionally, trials exhibiting abnormal pupil size measurements, where more than 50% of measurements were replaced by interpolation were excluded. For each participant, trials were further screened, removing those in which the average value exceeded four standard deviations from the mean pupil diameter (+4SD). Furthermore, due to the potential influence of low-level stimuli, particularly brightness on pupil size (Laeng and Sulutvedt, 2013), calculations for pupil size involved comparing pupil diameter done under our experimental conditions to baseline measurements (Mathôt et al., 2018). Thus, baseline pupil size was defined as the average pupil size within the 100 ms window before presentation (1.42 mm). Calibration was performed by subtracting the baseline pupil size from the measurement during stimulus presentation (corrected pupil size = pupil size − baseline), tailored to the specific requirements of eye tracking data integration.

### 2.5 EEG data preprocessing

**Figure 2:**
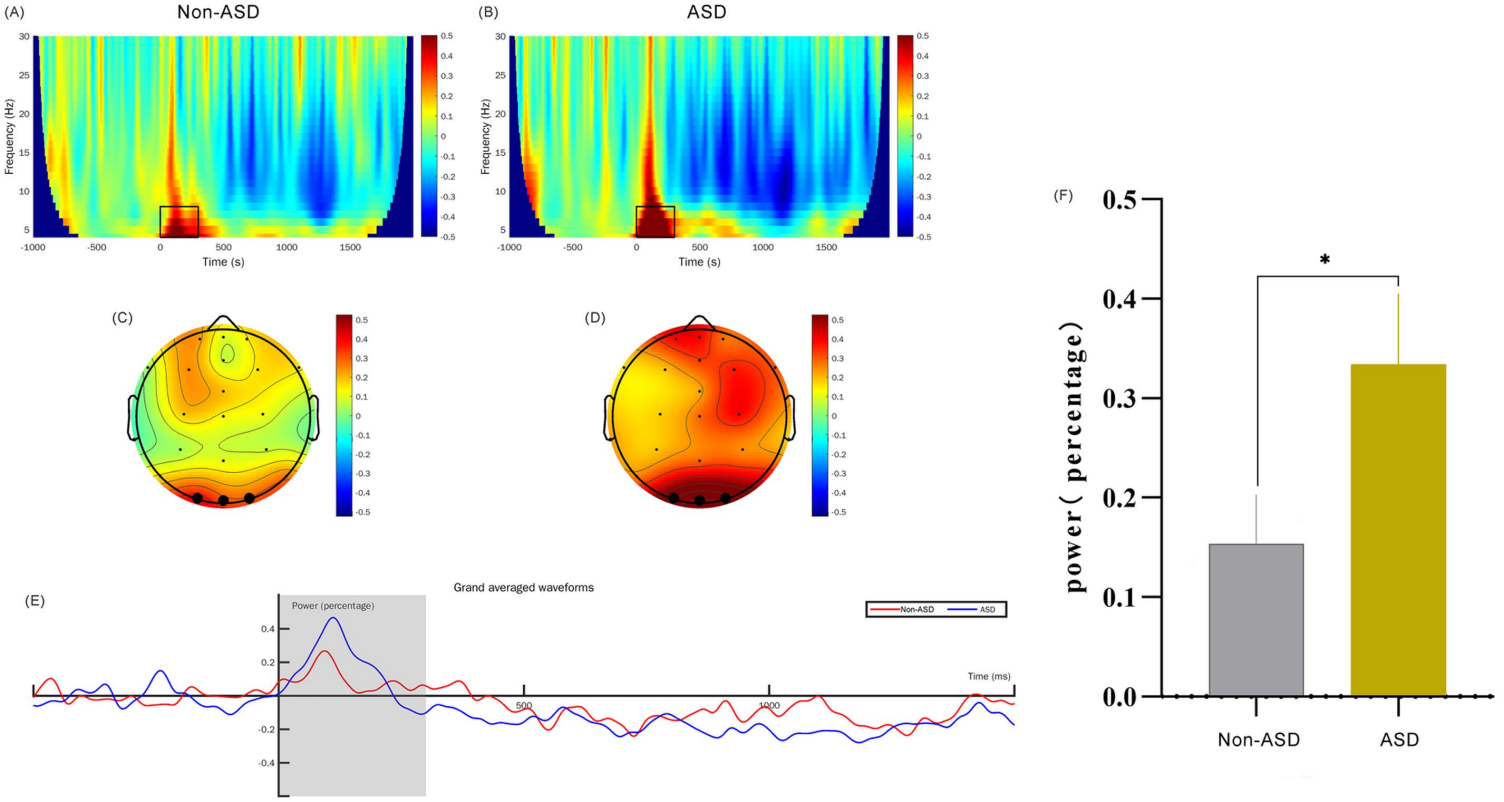
Comparison of *θ*-band time-frequency power for ASD and non-ASD Cohorts. (A,B) The time-frequency diagrams for the non-ASD and ASD groups respectively, and the selected time-frequency window is marked with the black box. (C,D) The time-frequency topographic maps of the non-ASD and ASD groups respectively, and the selected electrodes are marked in black. (E) The average time course diagram for the two groups showing the time-frequency power, and the shaded area is the 0–300 ms time window. (F) The comparison of the mean values of the time-frequency power between the two groups. “*” denotes *p* < 0.01.

**Figure 3:**
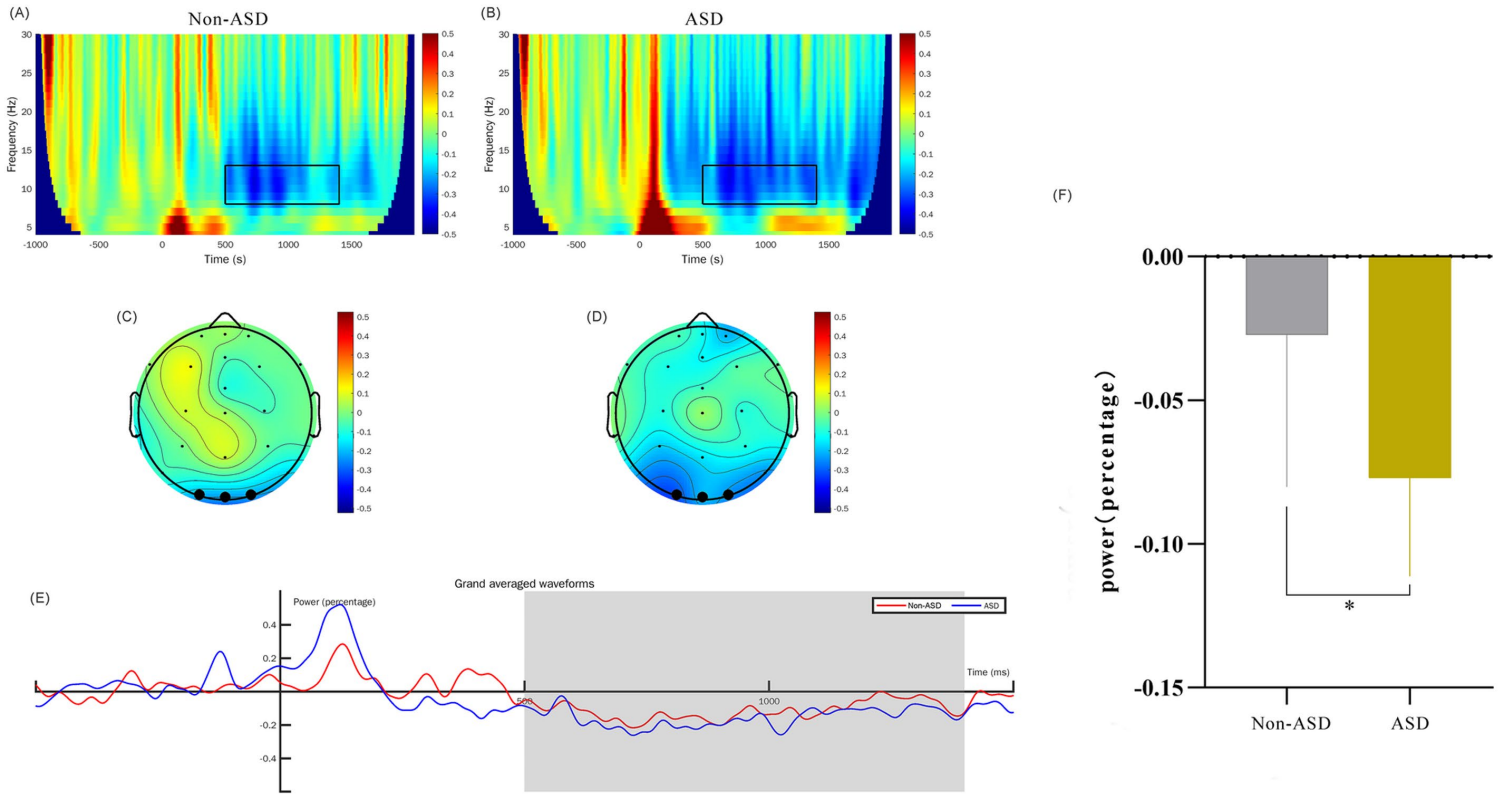
Comparison of *α*-band time-frequency power for ASD and non-ASD Cohorts. (A,B) The time-frequency diagrams for the non-ASD and ASD groups respectively, and the selected time-frequency window is marked with the black box. (C,D) The time-frequency topographic maps of the non-ASD and ASD groups respectively, and the selected electrodes are marked in black. (E) The average time course diagram for the two groups showing the time-frequency power, and the shaded area is the 500–1,400 ms time window. (F) The comparison of the mean values of the time-frequency power between the two groups. “*” denotes *p* < 0.01.

EEG data preprocessing was accomplished using the EEGLAB v.13.4.4b toolbox (see text footnote 2) under MATLAB 2019b (see text footnote 2). Firstly, we filtered the data by setting the lower edge of the frequency pass band to 0.1 Hz and the higher edge to 30 Hz, effectively eliminating any 50 Hz power frequency interference. This was followed by extracting epochs and correcting the baseline; we used the stimulation moment as the time origin and segmented the data into durations of −1 to 2 s. Next, we performed artifact rejection by dismissing the bad channels, leaving 22 electrodes remaining after interpolating electrodes. Independent component analysis (ICA) was then run to identify retained components. We further utilized the threshold method (ranging from −100 to 100 μV) to identify and eliminate any segments that exceeded the threshold. Finally, we reset the reference electrode to serve as the whole brain average reference electrode. We superimposed and averaged EEG signal segments evoked by restrictive interest paradigm and filtered these through a 30 Hz low-pass filter to yield event-related potential signals. The FieldTrip toolbox^3^ was then employed to carry out spectrum decomposition on the preprocessed −1 to 2 s segmented data. For this process, we selected the wavelet corresponding to the center frequency point of 3 cycles. The decomposition frequency was set to range from 4 to 30 Hz, with a frequency point interval of 1 Hz. The baseline (−624 to −376 ms) was corrected using the relchange method, our primary focus was on theta frequencies from 4 to 8 Hz at 0 to 300 ms (positive wave) and alpha frequencies from 8 to 13 Hz at 500 ms to 1,400 ms (negative wave), sourced from three electrodes in the occipital region (O1, Oz, O2). The results were visually represented through time-frequency maps and topographic maps, as detailed in Figures 2, 3. Supplementary File 1 contains further details on artifact rejection and cumulative data processing.

### 2.6 Statistical framework

Data analysis for this study was conducted using MATLAB2019b (see text footnote 2). Firstly, we performed a two-sample t-test to identify differences in developmental indicators between children with ASD and non-ASD children. This test also compared the differences in power in theta and alpha frequency bands in the occipital region and the differences in eye-tracking fixation time and pupil size when observing our 24-object interest paradigm. Secondly, Pearson correlation analysis explored the relationships among EEG time-frequency power, eye-tracking metrics, and developmental scores, and we calculated the area under the curve (AUC) of EEG and ET indicators (average fixation time and average pupil size) using receiver operating characteristic (ROC) curves. Finally, we used multivariate logistic regression analysis to establish a diagnostic prediction model that integrates these various parameters. Having age and gender as covariates, a comprehensive EEG time-frequency power, ET fixation time, and pupil size prediction model for ASD was established to identify potential biomarkers for the early diagnosis of ASD.

## 3 Results

### 3.1 Demographic and clinical characteristics

The study comprised a total of 59 children, with 32 in the ASD group and 27 in the age and gender-matched non-ASD group. The analysis revealed no significant differences in age (ASD: 3.1 ± 0.5 years, non-ASD: 2.9 ± 0.5 years; *p* = 0.493) and no gender disparity between the two groups (*p* = 0.191). The average developmental quotient (DQ) of ASD was (69.9 ± 3.6), which was considered mild developmental delay, while the average DQ of non-ASD children was (86.6 ± 5.4; *p* < 0.01). The mean CARS score for the ASD group was (32.9 ± 2.1) and the mean ADOS total score was (15.09 ± 3.0).

### 3.2 Overview of eye-tracking results

ET data showed differences in fixation time, and pupil size between ASD and non-ASD children during the viewing of the 24-object interest paradigm. Specifically, the data showed there were 9 key objects of interest: balloon, cake, cantaloupe, glasses, mail car, bullet train, plane, sailboat, and truck, as illustrated by the visual illustration of the ET results (Figure 1). We subsequently describe the measurements of fixation time and pupil size obtained via our eye-tracking paradigm.

### 3.3 Quantitative summary of fixation time outcomes

Fixation times for objects in the interest paradigm were compared between children with ASD and non-ASD children. Results revealed significant differences between the two groups across several objects of interest. Specifically, ASD children exhibited significantly greater mean fixation times compared to non-ASD children for the bullet train (*p* = 0.026), steamship (*p* = 0.031), sailboat (*p* = 0.037), sedan car (*p* = 0.041), and mail car (*p* = 0.047). However, non-ASD children had significantly greater mean fixation times compared to ASD children for balloon (*p* = 0.019), cap (*p* = 0.022), paper bag (*p* = 0.037), and cake (*p* = 0.017). Please refer to Table 1 for a detailed quantitative overview of the eye-tracking outcomes in terms of fixation time of the comprehensive list of 24 objects.

**Table 1:** Comparison of ET fixation time by object for ASD and non-ASD cohorts in milliseconds (ms).

	ASD		Non-ASD		*t*/*F*	*p-*value
	Mean	SD	Mean	SD		
Bullet train	9110.01	307.46	1689.12	569.03	2.34	0.026*
Steamship	8953.31	206.44	658.38	111.62	1.97	0.031*
Sailboat	8314.44	154.14	1812.71	249.39	1.61	0.037*
Sedan car	8104.97	324.91	2424.65	232.11	0.65	0.041*
Water bottle	6634.21	233.23	4434.06	220.26	0.27	0.207
Mail car	6214.02	615.49	176.31	193.03	1.52	0.047*
Truck	5510.06	673.85	3904.16	331.23	1.832	0.513
Cantaloupe	4478.39	252.59	4798.07	632.79	−1.31	0.199
Balloon	2011.75	134.65	10658.20	202.53	−2.33	0.019*
Electric-fan	1362.48	339.15	4654.11	373.42	−0.31	0.758
Plane	1340.32	293.01	0.00	—	—	—
Laptop	1278.19	361.74	0.00	—	—	—
Briefcase	1204.21	274.50	842.52	276.52	0.82	0.416
Trash can	1132.23	520.12	0.00	—	—	—
Bread	866.64	230.29	1658.18	124.61	1.10	0.276
Cap	552.45	216.94	6316.21	112.81	−1.22	0.022*
Paper bag	348.79	171.83	3502.49	144.26	−3.33	0.037*
Switch	298.15	81.06	0.00	—	—	—
Cake	274.81	120.24	6652.10	161.44	−3.18	0.017*
Glasses	110.06	94.04	316.24	86.93	−0.47	0.635
Mouse	0.00	—	3658.02	321.07	—	—
Umbrella	0.00	—	1538.65	261.03	—	—
Swivel chair	0.00	—	2812.18	115.02	—	—

A “0” means no object of interest/fixation time is less than 100 ms; “*” denotes *p* < 0.05 after Bonferroni correction.

### 3.4 Quantitative summary of pupillometry outcomes

The pupil sizes of children with ASD and non-ASD children were compared across the objects of interest during the viewing of the interest paradigm. Our results indicate significant differences in mean pupil sizes between the two groups across 9 objects. Specifically, compared to non-ASD children, ASD children exhibited greater mean pupil sizes for the bullet train (*p* < 0.001), sailboat (*p* < 0.001), mail car (*p* < 0.001), plane (*p* < 0.001), and truck (*p* < 0.001). However, compared to ASD children, non-ASD children demonstrated greater mean pupil sizes for balloon (*p* < 0.001), cake (*p* < 0.001), cantaloupe (*p* < 0.001), and glasses (*p* < 0.001). Please refer to Table 2 for a detailed quantitative overview of the eye-tracking outcomes in terms of pupil size of the comprehensive list of 24 objects.

**Table 2:** Comparison of ET pupil size by object for ASD and non-ASD cohorts in millimeters (mm).

	ASD		Non-ASD		*t*/*F*	*p*-value
	Mean	SD	Mean	SD		
Bullet train	4.03	0.31	1.53	0.12	0.97	<0.001
Steamship	1.44	0.22	1.19	0.07	0.37	0.701
Sailboat	4.48	0.37	1.09	0.11	3.42	<0.001
Sedan car	1.35	0.21	1.04	0.07	0.31	0.627
Water bottle	1.10	0.07	1.66	0.15	−0.27	0.611
Mail car	4.10	0.32	1.50	0.11	−0.51	<0.001
Truck	4.02	0.31	1.09	0.10	3.11	<0.001
Cantaloupe	1.35	0.12	3.03	0.32	−1.72	<0.001
Balloon	1.35	0.12	4.22	0.21	−0.39	<0.001
Electric-fan	1.50	0.11	1.26	0.11	0.40	0.522
Plane	3.31	0.29	1.09	0.14	1.84	<0.001
Laptop	2.47	0.12	0.00	—	—	—
Briefcase	1.33	0.10	1.19	0.08	0.23	0.461
Trash can	1.20	0.09	0.00	—	—	—
Bread	1.34	0.10	1.13	0.11	0.37	0.524
Cap	1.76	0.14	1.20	0.09	1.04	0.041*
Paper bag	1.54	0.07	1.41	0.17	0.47	0.722
Switch	1.13	0.04	0.00	—	—	—
Cake	1.39	0.11	3.21	0.21	−1.56	<0.001
Glasses	1.90	0.14	2.73	0.22	−2.96	<0.001
Mouse	0.00	—	1.17	0.11	—	—
Umbrella	0.00	—	1.41	0.26	—	—
Swivel chair	0.00	—	1.32	0.17	—	—

A “0” denotes the pupil is at the baseline value of 1.42 mm and indicates no change; “*” denotes *p* < 0.05 after Bonferroni correction.

### 3.5 EEG time-frequency analysis

We analyzed the EEG time-frequency responses with a particular focus on the theta (4–8 Hz) and alpha (8–13 Hz) frequency bands, examined within the 0–300 ms and 500–1,400 ms time windows, respectively. This analysis was conducted across the occipital region EEG channels (O1, O2, and Oz). To obtain evoked power, we calculated trial-averaged power by averaging the EEG signals across trials, which provided a robust measure of stimulus-related power changes. Baseline correction was applied from −624 ms to −376 ms to control for inter-trial variability. Our findings indicate that the theta band power in ASD children at 0–300 ms was significantly higher than that of non-ASD children (*p* < 0.01; Figure 2). Additionally, we observed that the alpha band evoked power in the occipital region at 500–1,400 ms was significantly different between groups (*p* < 0.05; Figure 3).

### 3.6 Predictive modeling using EEG indices

**Figure 4:**
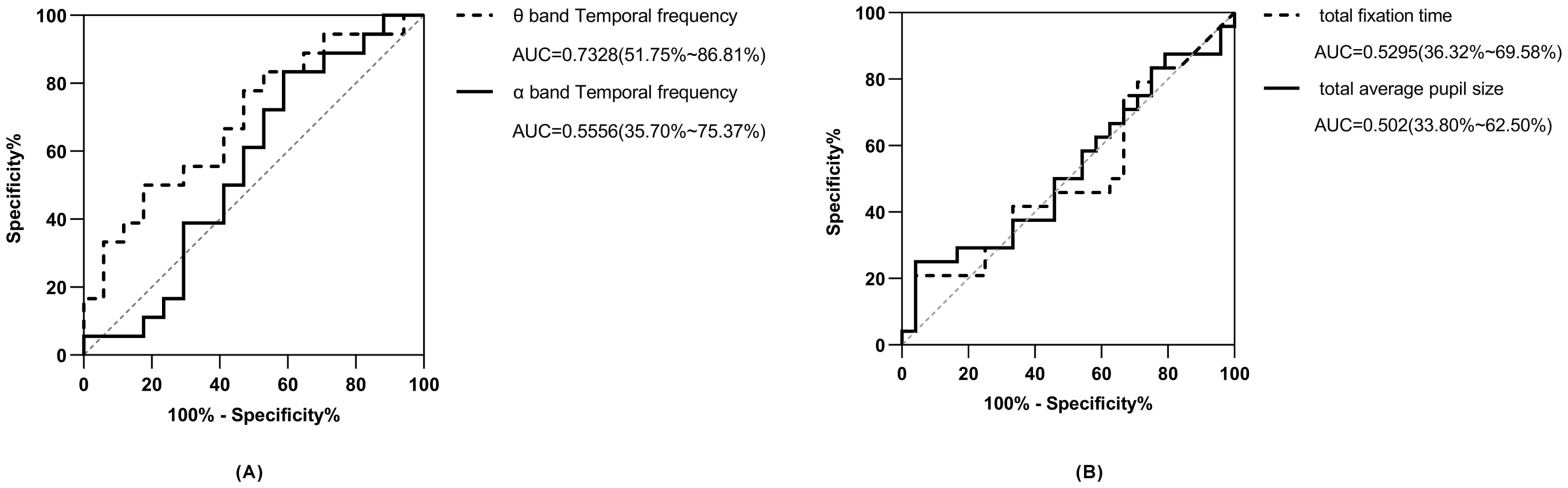
ROC curves of temporal frequency and pupil size indices. (A, black dotted line) ROC curve of time-frequency index in the theta band of the occipital lobe for predicting ASD. (A, black solid line) ROC curve of time-frequency index in the alpha band of the occipital lobe for predicting ASD. (B, black dotted line) ROC curve of eye tracking fixation time for index predicting ASD. (B, black straight line) ROC curve of eye tracking pupil size index for predicting ASD.

The area under the curve (AUC) for predicting ASD with the independent theta-band time-frequency power of the occipital lobe was 0.7328 (0.5175–0.8681), with a sensitivity of 77.78% and a specificity of 82.35%; the determined threshold for predictive power is 0.3000, as illustrated in Figure 4A. The AUC for predicting ASD with the independent alpha-band time-frequency power of the occipital lobe was 0.5556 (0.3574–0.7537) with a sensitivity of 55.56% and a specificity of 58.82%, and the cutoff was 0.0527, see solid line curve in Figure 4A.

### 3.7 Predictive modeling using ET indices

The AUC for predicting ASD phenotype using total eye-tracking fixation time alone was 0.53 (95% CI: 0.36–0.70) with a sensitivity of 54.2% and a specificity of 58.3%. The AUC for predicting ASD with total pupil size alone was 0.51 (95% CI: 0.34–0.67) with a sensitivity of 54.2% and specificity of 62.5% (Figure 4B).

### 3.8 Multi-modal predictive modeling

**Figure 5:**
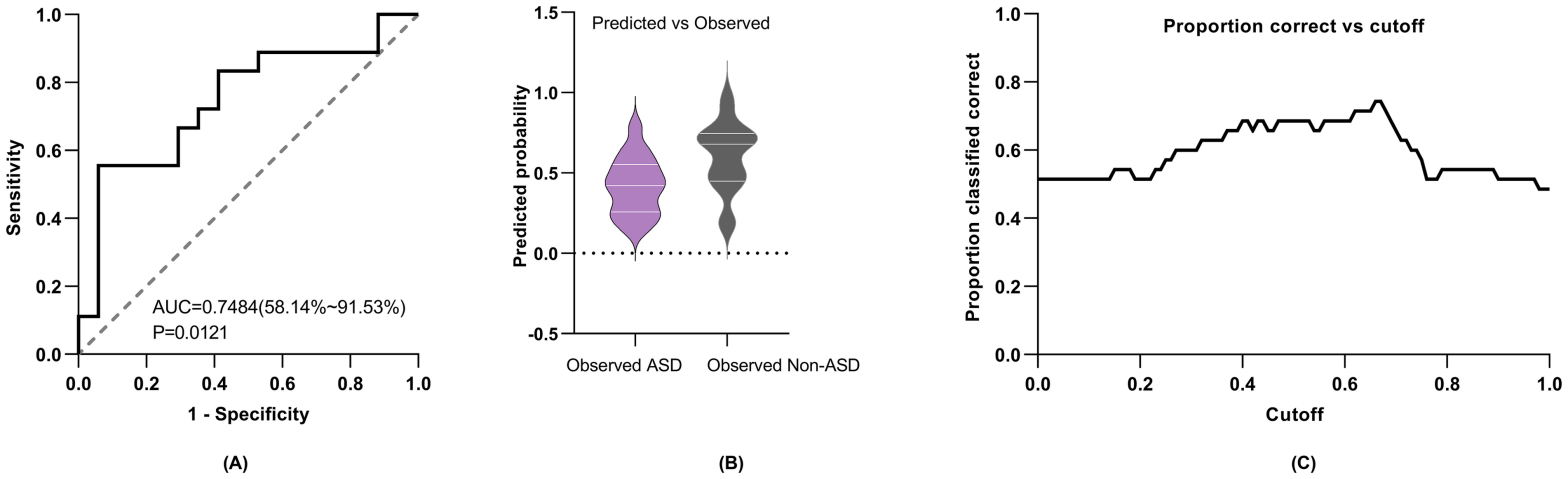
Diagnostic performance of multimodal logistic regression model. (A) ROC curve for multimodal model. (B) Model predicted and measured probabilities. (C) Cutoff value, with different prediction thresholds, the change in accurate prediction probability. “0” denotes ASD and “1” denotes non-ASD.

We constructed a multimodal logistic regression model incorporating ET fixation time, pupil size, EEG TF indices (*α* and *θ* power), gender, and age. This model, represented by the equation logit(P(Y = ASD)) = −1.581 + 0.2727 * age + 3.795 * *θ* band power − 1.515 * *α* band power − 0.2244 * gender − 1.613 * pupil size + 0.0001249 * total fixation time, demonstrated an Akaike information criterion (AIC) of 50.6. Additionally, the model achieved an area under the curve (AUC) of 0.75, with sensitivity at 66.7%, and specificity at 70.6%, using a cutoff value of 0.5 (Figure 5).

## 4 Discussion

Our study utilized a 2 4-object interest paradigm to explore the visual attention characteristics of children with and without ASD aged 2–4 years. Our findings highlight the tendency of children with ASD to have circumscribed interests, a component of RRBs, particularly oriented towards transportation-related objects. Notably, our results revealed differences in ET indices between ASD and non-ASD children. We observed significant increases in pupil size and fixation time (FT) among toddlers with ASD when viewing objects of restrictive interest (RI), and similarly observed these changes among toddlers without ASD when viewing neutral interest (NI) objects. To our knowledge, the current study is the first to study RIs in ASD using an integration of EEG TF power, pupil size, and fixation time with the aid of an object-only viewing paradigm.

Our results regarding changes in pupil size demonstrated that children with ASD had significantly increased mean pupil sizes for RI objects such as the mail car, bullet train, plane, sailboat, and truck, as compared to their non-ASD counterparts. This underscores the potential utility of pupil size as a marker for identifying restricted interest in ASD. To our knowledge, measuring changes in pupil size due to passive viewing of a 24-object interest paradigm has never been done before to identify circumscribed interests in ASD children. In recent years, studies have shown that changes in pupil size during viewing tasks may reflect psychological impairment, with abnormal changes in pupil size postulated as probable diagnostic and risk prediction indicator for autism, depression, schizophrenia, and other mental disorders (Kudinova et al., 2016; Nyström et al., 2018; Burley et al., 2019). Pupil measurements therefore offer an important avenue for gaining insights into the presence of RRBs, specifically restricted interests in ASD. Prior research has implicated the locus coeruleus-norepinephrine (LC-NE) system as an important underlying mechanism involved in attention regulation as measured by pupillometry. The LC-NE is posited to control attentional states, and it has been suggested that differences in attention capacities between ASD and non-ASD toddlers could explain pupil differences during the performance of tasks (Blaser et al., 2014; de Vries et al., 2021). Extending this idea to restricted interests could explain the differences in pupil size that we see in children with ASD compared to children without ASD, although the underlying reason for orienting towards transportation-related objects still remains unclear. Prior studies investigating circumscribed interests and pupillary changes in the ASD population have yielded diverse results. For instance, DiCriscio and Troiani (2017) found a correlation between pupil adaptation due to light stimulus and quantitative measures of ASD. Specifically, they found that pupil adaptation correlated with total Social Responsiveness Scale (SRS) score and most of its subscores, though the RRB subscore was unrelated to pupil size. Moreover, a separate study that examined the association between pupil light reflex (PLR) and autistic traits found that increased RRBs in adults were associated with weaker PLR, but this finding was not demonstrated in children (Soker-Elimaliah et al., 2023). Thus, given these evident disparities, we believe that instead of using light as a stimulus, using a passive viewing paradigm designed specifically for identifying restricted interests may offer a more effective means of differentiating ASD children from non-ASD children at a younger age based on changes in pupil size. Our novel interest paradigm thus offers this advantage over previous paradigms and tasks. We believe our results contribute to the growing evidence of using pupil size to identify traits of autism such as restricted interests and could help refine the development of early screening methods.

Furthermore, our utilization of FT proved successful in identifying circumscribed interests in ASD, with our findings being consistent with the current literature, where studies have noted an atypical preference for RI objects over NI objects among children with ASD (South et al., 2005; Sasson et al., 2008; Sasson and Touchstone, 2014; Frazier et al., 2017). In particular, our results resonated with a separate study conducted by Sasson and colleagues that also used eye-tracking technology and a visual exploration task involving object and social arrays to demonstrate patterns of attention in ASD and non-ASD children aged 2–5 years (Sasson et al., 2011). Similar to our results, they found that children diagnosed with ASD, relative to their non-ASD counterparts, exhibited prolonged fixation times for images associated with high autistic interest, such as trains, in contrast to objects with low autistic interest, such as clothing and food. This fixation on RI objects by ASD children could be explained by a visual preference for predictability. For example, a truck has a predictable purpose, as well as wheels that move in a repetitive and predictable motion, which has been posited to reduce anxiety in situations of hyper-arousal (Wang et al., 2018). Hence, the predictable nature inherent to the objects themselves, including the low-level properties of objects (e.g., wheels on a truck), explains the interest that children with ASD have for RI objects in our 24-object paradigm. The prolonged fixation times observed under restricted interest stimulation in our study may be a characteristic of ASD and thus, with more research, be considered when developing screening tools for RRB detection in ASD.

Interestingly, our 24-object interest paradigm was able to identify differential visual interest patterns in the non-ASD cohort. Our findings showed that non-ASD toddlers displayed increases in FT and pupil size in response to NI objects such as balloons, cake, cantaloupe, and umbrella. In a prior study using an object-only array, non-ASD children had no visual preference for any object (Sasson et al., 2008), which contrasts with our findings of circumscribed interests. An explanation for the non-ASD cohort demonstrating a preferential visual pattern can be attributed to several factors. Firstly, non-ASD children can also have restricted or intense interests at a certain age of development, and those interests often change or expand over time (DeLoache et al., 2007; Lewis and Kim, 2009; Burrows et al., 2021). Secondly, several of the NI objects that captured the interest of non-ASD children held social connotations, such as cake and balloons, commonly associated with celebratory occasions. Given the positive emotional associations linked with these objects, they likely elicited heightened attention from non-ASD children compared to other objects in the paradigm. Furthermore, increased interest in some objects could be due to prior exposure, such as the chair, fan, and cap. Studies have shown that differences in the amount of exposure to certain objects could drive differences in attentional preference and thus explain why non-ASD children were more interested in objects that are easily seen in daily life (Scheerer et al., 2021).

In response to viewing the 24-object interest paradigm, our study demonstrated a notable increase in TF power within the alpha and theta frequency bands at intervals 0–300 ms and 500–1,400 ms, respectively. This was specifically seen within the occipital region of the brains of toddlers diagnosed with ASD as compared to their non-ASD counterparts. Our results indicated that theta-band power was elevated in children with ASD during the early viewing period compared to non-ASD children, while alpha-band power was comparatively reduced. Baseline measurements showed differences between groups in theta but no significant difference in alpha waves. These findings may be due to differences in neural activity between the two groups at baseline. These differences may be partially or completely eliminated after the baseline correction, which more truly reflects the impact of interest stimulation on brain activity. The enhancement of theta activity in the ASD group may reflect attention control or attention distribution (Sauseng et al., 2007). Additionally, this increase may be due to heightened excitement in the cerebral cortex when viewing restricted-interest objects or may reflect a synchronous increase in neural network activity. Within the range of 500–1,400 ms, it was observed that alpha power in individuals with ASD was weaker than in non-ASD individuals. This may indicate a stronger response to viewing restricted-interest objects or suggest challenges in processing information. The lack of inhibitory activity in the cerebral cortex may make it difficult for individuals with ASD to effectively integrate and filter unrelated information, which can impact normal social functioning. Of course, verification of these findings through the use of big data is necessary.

In a cross-sectional replication study of 143 infants with varying familial risk for ASD, Haartsen et al. demonstrated that having an increased alpha connectivity at 14 months predicts a subsequent diagnosis of ASD and the development of RRBs, with restricted interests having the strongest association (Haartsen et al., 2019). Moreover, they also proposed that abnormalities seen in the frontal and striatal structures of the brain contribute to the manifestation of restricted interests among children with ASD. This idea of structural or functional brain aberrations has been seen across several studies that report cortical thickening of the corpus callosum during infancy and its higher structural connectivity with the cerebellum at 6 months being linked to the subsequent development of higher order RRBs such as circumscribed interest. According to Wolff et al. (2017), these findings support the idea that increased alpha connectivity within these aberrant neural pathways are indicative of an over-focused attention style most strongly associated with restrictive interests.

Although interesting, it is crucial to note that there is no consensus in the research community regarding these findings. Several studies have reported an increase in alpha band power (Sutton et al., 2005; Chan and Leung, 2006; Cornew et al., 2012) in individuals with ASD, while others have noted a decrease (Sheikhani et al., 2012; Afifi et al., 2015; Takagaki et al., 2015) or found no significant difference (Hyvärinen and Oja, 2000; Coben et al., 2008). These findings align with a systematic review by Bogéa Ribeiro and da Silva Filho (2023) that showed significant variations in the alpha frequency band, with TF power being reduced in children of the same age and elevated in studies involving older children (Machado et al., 2015). The discrepancies witnessed across studies can be attributed to factors such as varying sample sizes, the age range of subjects, and differing experimental approaches. In addition, these differences may also underscore the complexity and heterogeneity of ASD, with studies possibly reflecting different subgroups within the ASD population and varying developmental trajectories. Several theories have been proposed to account for these differences in power across various frequency bands. One such hypothesis is the U-shaped profile theory, which posits that individuals with ASD may demonstrate excessive power in both the lower (delta and theta) and higher (beta and gamma) frequency bands while showing diminished power in the intermediate alpha band (Wang et al., 2013). Our findings suggest an atypical pattern of neuronal activity in the occipital region of ASD toddlers when engaged with objects of high autistic interest. It lends further credence to our hypothesis that when visually stimulated with images of high interest associated with ASD, toddlers with this disorder exhibit different brain activity patterns compared to their non-ASD counterparts. These observed differences in neuronal activity between ASD and non-ASD toddlers indeed lend support to the functional underpinnings and heterogeneity inherent to ASD (Masi et al., 2017). It underscores the theory of local over-connectivity in various brain regions, a phenomenon associated with ASD (Happé and Frith, 2006; Klimesch et al., 2007). Thus, over-connectivity in the occipital brain region of ASD toddlers may explain the pattern of neuronal activity identified relating to increased power across the alpha and theta frequency bands, highlighting the complexities and individual differences present within ASD as a condition.

In order to better understand the relationship and significance of ET and EEG indices in relation to circumscribed interest in autism, we constructed multiple univariate logistic regression models to explore these associations. Our regression model utilizing the independent *θ* band TF power of the occipital lobe proved to be superior in predicting ASD, achieving an AUC of 0.73 (95% CI: 0.52–0.87), with a sensitivity of 0.78 and a specificity of 0.82, much better than the univariate models which were mostly slightly above 0.5 [univariate models which utilized *α* band TF power, total eye fixation time, and total pupil size, with AUCs of 0.56 (95% CI: 0.36–0.75), 0.53 (95% CI: 0.36–0.70) and 0.50 (95% CI: 0.34–0.67) respectively]. The superior predictive capacity of the θ band index in predicting ASD as an outcome could potentially be elucidated by the localized cortical overconnectivity of neuronal circuits within the occipital lobe, whereas *α* oscillations are indicative of and rely more heavily on long-range, global connections across brain regions (Nunez and Srinivasan, 2006). Recent autism research has explored the integration of ET and EEG modalities to predict ASD as an outcome, with the majority employing paradigms centered around social or facial stimuli (Thapaliya et al., 2018; Geng et al., 2020; Zhang et al., 2021). To the best of our knowledge, employing a multimodal approach to investigate circumscribed interests in ASD through an object-only paradigm, with the integration of both EEG and ET indices represents a novel endeavor that has not been previously undertaken. When integrating both ET and EEG indices into our multivariate regression model, while adjusting for age and gender, we observed a notable improvement of the AUC which was 0.75 for predicting ASD. The improvement in model’s performance compared to our best performing univariate *θ* band model can be credited to the incorporation of both modalities into the regression analysis. This highlights the effectiveness of ET and EEG as tools not only for detecting restricted interests in ASD toddlers but also for identifying potential biomarkers for early ASD diagnosis, preceding the manifestation of behavioral traits. Furthermore, the utilization of EEG and ET, especially in clinical settings, is justified by their user-friendly nature, affordability, and time-saving benefits (Gurau et al., 2017; Thapaliya et al., 2018), in addition to their potential for early diagnosis.

While our study yielded promising results in objectively identifying RRBs in toddlers with ASD, we also encountered several limitations. Firstly, one limitation was the narrow age range of our cohort, which only included children 2–4 years old. Studies have shown that children acquire RRBs and restricted interests at different time points during development, with often “lower order” behaviors such as stereotyped behaviors manifesting in younger children and “higher order” behaviors such as restricted interests manifesting in older individuals (Lewis and Kim, 2009). Given the dynamic nature of RRBs and specifically restricted interests across development, future studies may benefit from employing longitudinal approaches or including a broader age range of participants to better capture these complexities. Another notable limitation was the small sample size of both the ASD and non-ASD cohorts in the current study, which hindered our ability to effectively internally validate our models’ results. This constraint was further compounded by the fact that we had no independent dataset to use for external validation. Therefore, future endeavors should prioritize replicating studies of this nature with larger cohorts and an independent dataset to ensure the comprehensive validation of the results obtained. Furthermore, the ethnic makeup of our cohorts was predominantly Chinese, potentially limiting the generalizability of our findings to other populations with a more heterogeneous ethnic makeup. It is important to note the lack of intelligence quotient (IQ) matching of participants in our current study. Nonverbal IQ in children with ASD has been found to be positively correlated with circumscribed interests (Gabriels et al., 2005), thus supporting the need to have IQ-matched ASD and non-ASD subjects.

## 5 Conclusion

This study sheds light on the eye-tracking patterns and EEG characteristics associated with restricted interests in children with ASD, viewed through a developmental lens. Each metric—eye-tracking fixation time, pupil size, and EEG theta and alpha power—holds promise as a potential biomarker for early ASD identification, offering insights into the neural mechanisms underlying restricted interests in autism. Notably, our logistic regression model, which integrates ET and EEG metrics, provides strong evidence supporting a multimodal approach to early ASD detection. This complementary assessment strategy could enhance detection accuracy, especially when used in conjunction with traditional diagnostic methods. Future studies will aim to further validate the sensitivity and specificity of these biomarkers and investigate their effectiveness across diverse age groups and demographics.

## Data Availability

The raw data supporting the conclusions of this article will be made available by the authors, without undue reservation.

## Footnotes

^1^https://www.adobe.com/cn/products/photoshop.html

^2^https://sccn.ucsd.edu/eeglab/index.php

^3^https://www.fieldtriptoolbox.org/
