# Supplementary material for: Interest paradigm for early identification of autism spectrum disorder: an analysis from electroencephalography combined with eye tracking
Source: Front Neurosci. 2024 Nov 27;18:1502045. doi: 10.3389/fnins.2024.1502045 (PMC11631861; doi:10.3389/fnins.2024.1502045)
Supplement: Supplementary file 1 [file Data_Sheet_1.docx]

**Supplementary File 1**

**Restricted interest task process**

The experiment began with a five-point calibration of the eye-tracking instrument to ensure accuracy. Following calibration, a 10-second blank screen served as a baseline, after which a fixation cross (“+”) appeared for a 1-second interval. Subsequently, a restricted interest image (see Figure 1) was displayed for 10 seconds, followed by the fixation cross. This sequence of the fixation cross and restricted interest image was repeated five times, resulting in a single experimental session lasting approximately 65 seconds. After each session, participants rested for 1-3 minutes, depending on their readiness, before starting a second session. The entire experimental process lasted between 2 and 5 minutes.

For Table 1, the maximum gaze time of 10 seconds represents the average gaze duration across 10 trials for each participant over two sessions. EEG data were collected across both sessions, resulting in 10 cumulative trials per participant.

**Correlation between EEG time-frequency results and developmental level**

During the interest paradigm, theta-band power (4-8 Hz) in the occipital region of children with ASD at 0-300 ms showed a negative correlation with adaptability scores (R=0.4683, P=0.045), whereas no significant correlation was observed in healthy control (HC) children (R=-0.1091, P=0.6768; Figure S1). Additionally, the phase-locked power in the alpha frequency band (8-13 Hz) at 500-1400 ms was negatively correlated with adaptability (R=-0.4846, P=0.0416) and showed a weak positive correlation with the CARS score (R=0.2076, P=0.0485) in the ASD group (Figure S2). No significant correlations were found in the HC group.


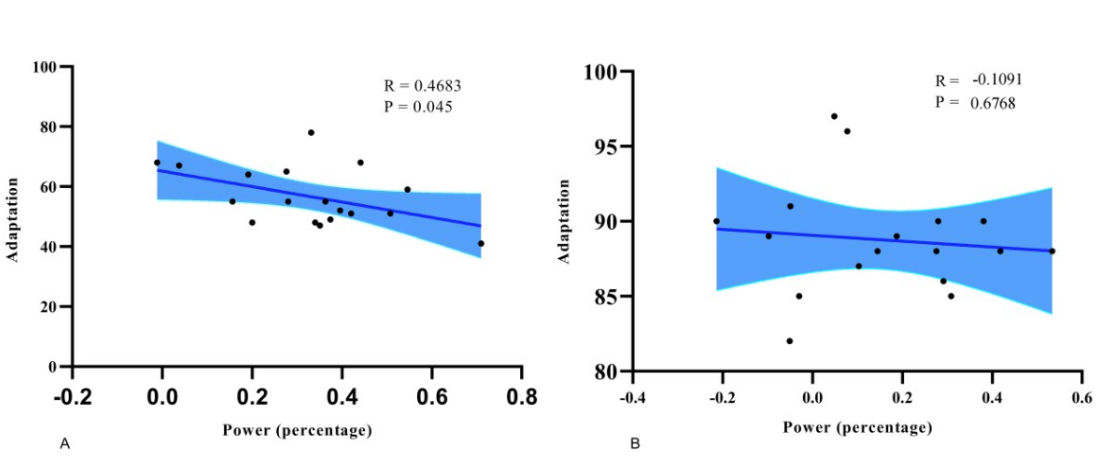


**Figure S1.** Correlation Between EEG Theta Power and Developmental Adaptability in ASD.


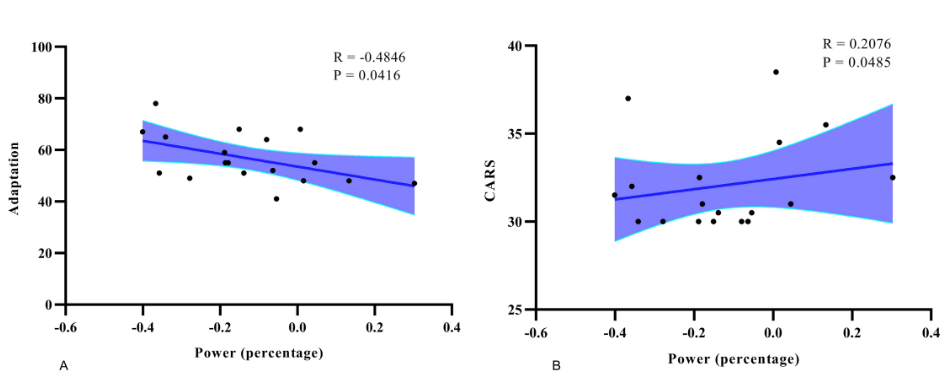


**Figure S2.** Correlation of EEG Alpha Power with Adaptability and CARS Scores.

**Correlation between EEG time-frequency results between pupil size**

We examined the relationship between pupillary responses to nine key objects of interest (balloon, cake, cantaloupe, glasses, mail car, bullet train, plane, sailboat, and toy train) and EEG time-frequency results (Figure S3). In children with ASD, a positive correlation was found between theta-band power (4-8 Hz) in the occipital region at 0-300 ms and pupil size for the bullet train (R=0.4685, P=0.0499), while no significant correlation was observed in typically developing (TD) children. Additionally, the phase-locked power evoked in the alpha frequency band (8-13 Hz) at 500-1400 ms was positively correlated with the pupil size for the sailboat (R=0.2375, P=0.0402) in the ASD group, with no significant correlation in the HC group. For the remaining seven objects of interest, no significant relationships were identified between pupillary indicators and EEG time-frequency results in either group.

**Figure S3.** Correlation of EEG Time-Frequency Power with Pupil Size in ASD.
