# Supplementary material for: Interest paradigm for early identification of autism spectrum disorder: an analysis from electroencephalography combined with eye tracking
Source: Front Neurosci. 2024 Nov 27;18:1502045. doi: 10.3389/fnins.2024.1502045 (PMC11631861; doi:10.3389/fnins.2024.1502045)
Supplement: Supplementary file 2 [file Data_Sheet_2.docx]

**Supplementary File 2**

**Information on artifact rejection and EEG preprocessing**

The purpose of running ICA here was to remove artifacts and separate signals. Through ICA, EEG signals could be decomposed into multiple independent components to identify and eliminate artifacts caused by non neural signals (such as eye movements, muscle activity, heartbeat, etc.), as well as separate and preserve useful EEG activity signals.

**The characteristics of electrooculography artifacts**: concentrated in leads such as Fp1 and Fp2 that were close to the eye position, with power concentrated at low frequencies (<5Hz)The electrooculography signal was similar to a step function

Blink artifact characteristics: concentrated in the frontal area and the power was concentrated in the low frequency range, with a step like waveform

**Eye movemont artifact characteristics**: concentrated on the frontal side, with topographic maps of positive and negative power on the left and right sides, and power concentrated in the low-frequency range

**Characteristics of electromyographic artifacts**: Power was concentrated at high frequencies (20Hz and above), located outside or very close to the skull on EEG maps. **ECG artifact characteristics**: around 1Hz Topographic map with linear gradient.

**Power frequency nois**e: peak power spectrum at 50Hz or 60HZ

**Channel noise:** Noise caused by poor channel contact and electrode sliding, almost all of which are concentrated in one channel and usually do not affect other channels. Features: Localized topographic map, consistently fluctuating or highly amplitude curves, decreasing power spectrum

**Others:** they did not belong to any of the above artifacts and may be a mixture of EEG and other artifacts, mainly manifested as patchy or scattered topographic maps.
